# The effect of the stromal component of breast tumours on prediction of clinical outcome using gene expression microarray analysis

**DOI:** 10.1186/bcr1506

**Published:** 2006-06-21

**Authors:** Susan J Cleator, Trevor J Powles, Tim Dexter, Laura Fulford, Alan Mackay, Ian E Smith, Haukur Valgeirsson, Alan Ashworth, Mitch Dowsett

**Affiliations:** 1 Breakthrough Breast Cancer Research Centre, The Institute of Cancer Research, Fulham Road, SW3 6JB, London, UK; 2 Current address: Department of Oncology, St. Mary's Hospital, Praed Street, London W2 1NY, UK; 3 Parkside Hospital, Parkside, SW19 5NX, London, UK; 4 Breast Unit, Royal Marsden Hospital, Fulham Road, London, SW3 6JJ, UK; 5 Department of Academic Biochemistry, Royal Marsden Hospital, Fulham Road, SW3 6JJ, London, UK

## Abstract

**Introduction:**

The aim of this study was to examine the effect of the cellular composition of biopsies on the error rates of multigene predictors of response of breast tumours to neoadjuvant adriamycin and cyclophosphamide (AC) chemotherapy.

**Materials and methods:**

Core biopsies were taken from primary breast tumours of 43 patients prior to AC, and subsequent clinical response was recorded. Post-chemotherapy (day 21) samples were available for 16 of these samples. Frozen sections of each core were used to estimate the proportion of invasive cancer and other tissue components at three levels. Transcriptional profiling was performed using a cDNA array containing 4,600 elements.

**Results:**

Twenty-three (53%) patients demonstrated a 'good' and 20 (47%) a 'poor' clinical response. The percentage invasive tumour in core biopsies collected from these patients varied markedly. Despite this, agglomerative clustering of sample expression profiles showed that almost all biopsies from the same tumour aggregated as nearest neighbours. SAM (significance analysis of microarrays) regression analysis identified 144 genes which distinguished high- and low-percentage invasive tumour biopsies at a false discovery rate of not more than 5%. The misclassification error of prediction of clinical response using microarray data from pre-treatment biopsies (on leave-one-out cross-validation) was 28%. When prediction was performed on subsets of samples which were more homogeneous in their proportions of malignant and stromal cells, the misclassification error was considerably lower (8%–13%, *p *< 0.05 on permutation).

**Conclusion:**

The non-tumour content of breast cancer samples has a significant effect on gene expression profiles. Consideration of this factor improves accuracy of response prediction by expression array profiling. Future gene expression array prediction studies should be planned taking this into account.

## Introduction

Breast tumours are routinely subclassified according to microscopic morphology, immunohistochemical staining, and stage. On the basis of this clinical information and patient age, an estimate of prognosis may be derived [1,2]. Most clinicians make recommendations regarding the need for adjuvant chemotherapy on the basis of this estimate.

However, breast cancer is a heterogeneous disease, and differences in prognosis and response in distinct molecular subgroups need to be taken into account. Improvement in the accuracy of prediction of prognosis without systemic treatment or with endocrine treatment alone would allow avoidance of non-beneficial chemotherapy in a significant proportion of women [3]. Additionally, experience with neoadjuvant chemotherapy has demonstrated resistance in a significant proportion of primary breast tumours [4]. These patients derive no downstaging benefits from neoadjuvant chemotherapy. Furthermore, chemosensitivity in the neoadjuvant setting is associated with superior long-term survival (relative to chemoresistance) [5] and therefore may represent a marker of survival benefit from chemotherapy. Identification of a chemoresistant profile would allow further tailoring of treatment by enabling selection of tumours unlikely to respond and therefore unlikely to derive a survival benefit.

Several studies have demonstrated that gene expression microarray profiling may be useful in improving prediction of prognosis [6-9] and treatment response [10-16]. These studies employed non-dissected surgical [7-9], core-cut biopsy [12], or FNA (fine needle aspiration) samples [10]. However, breast tumours are non-homogenous in nature. They include inflammatory and vascular elements but most significantly (by proportion) connective tissue components [17]. The proportions of these components vary according to tumour type and sample type and also across a single tumour [17]. In studies involving surgical samples, those used for profiling can be selected as those with the highest proportional malignant cell content. In studies involving biopsies, this is not possible and the researcher is required to set an arbitrary minimum percentage tumour limit.

The impact on expression profile of variation in the proportion of tumour cells and the nature of the non-tumour components have been largely unexplored. In this paper, we examine the effect of percentage tumour content on expression profile within a study designed to derive an expression profile predictive of response to adriamycin and cyclophosphamide (AC) neoadjuvant chemotherapy. We also consider methods for improvement of molecular profile-based prediction of response to primary chemotherapy by classification of samples according to cellular makeup or by the incorporation of sample tumour content information into the predictor.

## Materials and methods

### Patients and samples

Patients were recruited and treated at the Royal Marsden Hospital (RMH), London, UK. Eligible patients were those undergoing neoadjuvant AC chemotherapy treatment at doses of 60 and 600 mg/m^2^, respectively, three times a week, for a clinically measurable breast tumour. The study was approved by the RMH Clinical Research and Ethics Committees (study number. 1,947), and written consent was obtained in all cases. Patients had been allocated neoadjuvant treatment for one of several standard indications, including locally advanced or inflammatory breast cancer, high tumour-to-breast size ratio, and tumours located close to the nipple.

Diagnosis was confirmed histologically by core-cut biopsy. All patients on hormone replacement therapy at diagnosis were advised to discontinue this treatment. Patients who demonstrated at least a partial clinical response received six cycles of treatment prior to local treatment. Patients in whom there was no or only marginal response after three or four cycles proceeded directly to local treatment or were commenced on alternative systemic treatment (docetaxel).

Clinical size of tumour (largest diameter and a diameter perpendicular to this) was recorded prior to commencement and at completion of treatment. Clinical response was categorised as follows: no palpable abnormality after treatment, complete clinical response (cCR); more than 50% reduction in the product of the bidimensional measurements, partial response (PR); less than 50% reduction in the product of bidimensional measurements was recorded as no change (NC); and residual ill-defined thickening after a good response, minimal residual disease (MRD). Those cases in which there was no residual invasive carcinoma at surgery were classified as a complete pathological response (pCR). Good responders were defined as pCR, cCR, or MRD; poor responders were defined as PR or NC. These categories were chosen on the basis of our previous study, which showed that patients with 'good' response had superior overall survival relative to those with 'poor' response [18]. A proportion of patients undergoing a complete clinical and radiological (on ultrasound) response received radiation only as local treatment. Therefore, some of the cCRs may represent undocumented pCRs.

Research 14-gauge core biopsies were collected prior to commencing treatment and snap-frozen in liquid nitrogen. When consented to, a repeat sample was taken at 21 days after the first cycle of chemotherapy. All samples were thereafter coded using a study number as an identifier. Frozen cores were embedded in OCT (optimum cutting temperature embedding compound) and sectioned at -20°C in a cryostat. Sections (5 μm in thickness) were taken for haematoxylin and eosin staining to assess histological character superficially from the core as soon as 'full-face' was reached. The percentage of cells comprising invasive malignant disease and non-malignant components (that is, *in situ *disease, inflammatory infiltrate, non-malignant ductal/lobular structures, and fibroblastic involvement) were recorded by consensus between two breast pathologists. For patients in whom multiple biopsies were available, the biopsy with the highest invasive content was used for microarray analysis. Biopsies with less than 20% invasive cancer content were excluded from the study.

### RNA extraction and amplification

Cores were extracted from OCT as described by Ellis and colleages [19] and pulverised using a pestle and mortar on a bed of dry ice and subsequently in 1 ml of Trizol reagent (Invitrogen, Carlsbad, CA, USA) with a 'Polytron' homogenizer. Standard 'Trizol' RNA extraction was carried out without use of a carrier according to the manufacturers' instructions. Samples not giving distinct 18S and 28S peaks on an Agilent Bioanalyzer (Agilent Technologies, Palo Alto, CA, USA) trace were excluded from the study. Multiple cores from the same patient were handled separately for RNA extraction. A single round of T7 linear RNA amplification was carried out using the RiboAmp kit (Arcturus, Sunnyvale, CA, USA) with a starting amount of 1 μg when available or 50% of the available RNA.

Reference RNA was generated from a pool of RNAs extracted from 20 independent breast cancer surgical samples.

### cDNA array hybridisation

Microarray analyses used in-house (Breakthrough Breast Cancer Research Centre, London, UK) human arrays spotted with DNA derived from 4,600 IMAGE cDNA clones in duplicate. This set of genes represents a subset of the 5,808 Cancer Research UK (London, UK) gene set that was designed to include a high proportion of genes documented as being involved in carcinogenesis or tumour biology. To improve 'coverage' of genes involved in breast cancer, a list of discriminatory genes cited in important microarray studies on clinical breast cancer samples at time of production was compiled [7,20-22], and the array was supplemented with these. One half to two micrograms of sample RNA and a matched amount of reference RNA was labeled using the Powerscript Labeling kit (Clontech, Mountain View, CA, USA) in combination with Amersham Cy dyes (GE Healthcare, Little Chalfont, Buckinghamshire, UK). A single dye swap experiment was performed for each clinical sample. Slides were scanned using a GenePix 4000B (Axon Instruments Inc., Union City, CA, USA) scanner and GenePix version 4.0 software.

### Data analysis

Most of the data analyses were carried out with the S-plus statistical software package (Insightful, Seattle, USA) and purpose-written scripts (T Dexter, Breakthrough, UK). Raw expression values were transformed to Log_2 _ratios (sample/reference). The loess function [23] was used to remove biases due to the spot position and spot intensity. Flagged spots were treated as missing values. Log ratio values from duplicate spots and hybridisations were averaged. Genes with consistently low intensity and those that exhibited little variation across samples were removed from the analysis. After the above pre-processing, 1,286 genes remained for the prediction analysis. Samples were clustered both by complete linkage and flexible beta (beta = -0.5) agglomerative algorithms with (1 - correlation) as a distance measure [24,25]. The correlations were estimated using Spearman's rank method.

The nearest neighbour class prediction algorithm (euclidean distance) was used for all classifications because of its simplicity and good performance on microarray data as reported by Dudoit and Fridlyand [26]. We elected to use seven nearest neighbours throughout to give more stable error estimates and greater robustness than would result with smaller neighbourhoods. The weighted Kolmogorov-Smirnov statistic was used to rank genes for discriminatory information [27]. Each predictor was built starting with the highest ranked two genes from the training set, and then genes were added to the predictor in decreasing rank order until the error rates no longer decreased.

Owing to the limited number of samples, the misclassification error was estimated by leave-one-out cross-validation (LOOCV). In this approach, the class (that is, response) of each sample was predicted in turn, using the other samples as the training set. To avoid selection bias, the genes that were used as predictors were re-selected for each of these leave-one-out classifications [28]. To estimate the probability of the misclassification error arising by chance, a permutation *p *value was determined as suggested by Radmacher and colleagues [28]. In this procedure, the LOOCV estimate was determined 1,000 times for permuted class labels; the fraction of these that gave an equal or lower error estimate than that with true labels is taken as the *p *value. We refer to the latter as a 'label permutation'.

To assess the significance of lower misclassification error estimates for selected subsets of the samples, we needed to control for the smaller sample sizes. To this end, we defined a 'subset permutation' *p *value as the fraction of random subsets (1,000 matched for size and class proportions) of the full set of samples that gave rise to LOOCV error estimates equal to or lower than the selected subset. The correct class labels were used for subset permutation. The minimum error for each permutation across the top 2 to 10 ranked genes was used to calculate the *p *values for both of the above types of permutation to avoid bias. Although this is an arbitrary range, the minimum error rates in all the permutations had increased by a cutoff of 10. Significance analysis of microarrays (SAM) analyses were performed using standard software [29]. Pre-treatment samples only were used for prediction analyses.

## Results

### Patient and tumour characteristics

RNA of adequate amount and quality was available from 43 tumours before treatment. Of these, 23 (53%) demonstrated a 'good' and 20 (47%) a 'poor' clinical response. For 16 of these tumours, a paired 21-day sample was also available. These 'on treatment' biopsies are included in the analysis of reproducibility shown below to increase the number of paired samples but are excluded from the analysis of response prediction. The good responses comprised 16 (37%) that underwent a cCR, three (7%) that exhibited ill-defined thickening (MRD) at the end of treatment, and four (9%) that underwent a pCR (all cCR or MRD). The patient and tumour characteristics are shown in Table 1. The only feature differing between good and poor responders was tumour size, with pre-treatment size greater in poor compared with good responders (Mann-Whitney, *p *= 0.03). Pre-treatment size did not relate to expression profile (data not shown).

### Biopsy characteristics

A total of 147 cores were sectioned in the course of this study, including 104 that were sectioned at three levels (levels approximately 50 μm apart). Of these 104 cores, only 16 cores showed more than 10% absolute variation in invasive tumour content across all three sections and only one core showed more than 20% variation. Only four cores were found to have tumour at some levels and none at others; all four contained not more than 15% invasive tumour at the lowest level and were therefore excluded from the study. This suggests that the histological composition did not vary widely over the width of the core. The histological result from the lowest section for a given biopsy was taken as that most representative of the remaining biopsy used for profiling and hence the level on which percentage invasive tumour was assessed. The distribution of percentage invasive content of core (in cases of multiple cores, that core used for prediction analysis) did not relate to response as assessed by Wilcoxon rank sum test (*p *= 0.3) or *t *test (*p *= 0.27).

Percentage invasive content in core biopsies included in the prediction study varied from 20% to 95% (median 50%). For most core biopsies, the majority of the non-malignant tissue consisted of connective tissue. Inflammatory infiltrate was also scored on the lowest section as nil, mild, moderate, or severe. Of all 147 core biopsies, only six (4%) were scored as having a 'severe' inflammatory infiltrate at one or more levels.

### Basic validation of array data

To assess consistency of the expression profile between repeated biopsies, the 43 pre-treatment and 16 paired post-treatment samples were clustered (Additional file 1). The post-treatment samples were included in this validation study only to enhance numbers and were not used in subsequent predictive analyses. All but one pair (b223A and B) of duplicate biopsies taken at the same time point relative to treatment (in total, five pre-treatment and five post-treatment pairs) clustered as nearest neighbours despite some variation in percentage tumour between the pairs (Additional file 3). Of 16 pre/post-treatment pairs, 14 clustered with samples from the same tumour (Additional file 1). To validate our class prediction methodology, a supervised analysis was undertaken to obtain a gene list for prediction of oestrogen receptor (ER) status in pre-treatment samples (ER-α was excluded from the analysis). ER status was correctly assigned in 40 of 42 cases on LOOCV. Discriminatory genes are listed in the supplementary information (Additional file 4).

### Effect of percentage tumour on expression profile

Before performing prediction analysis, an exploratory analysis was undertaken to assess the impact of variation in the histological content of biopsies on the expression profile. A SAM regression analysis was performed to establish whether it was possible to identify genes that correlated with the proportion of tumour cells in the 43 pre-treatment core biopsies. One hundred forty-four genes were significant at a false discovery rate of not more than 5%. Table 2 shows the most correlated genes from this analysis. Positive values for 'score' (bold) indicate positive correlation with high percentage tumours, and negative values (underlined) indicate negative correlation with proportion of tumour cells in the samples. More genes correlated positively with the stromal content (*n *= 128) than with the tumour content (*n *= 16), possibly reflecting the greater molecular heterogeneity of tumour types across the samples than that of their associated stromas. This is also reflected in the higher false discovery rates for 'tumour-associated genes'.

### Response prediction using pre-treatment biopsies

**Figure 1:**
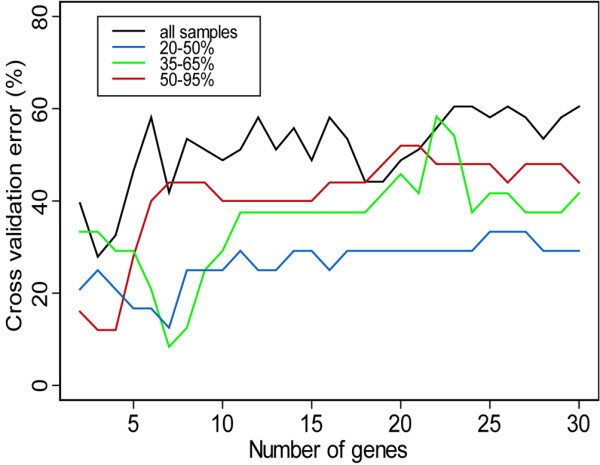
Leave-one-out cross-validation (LOOCV) error rates for response prediction. Variation in the LOOCV error estimate with the number of genes used in the predictor. Misclassification rates are plotted for the whole data set (*n *= 43) and subsets according to percentage tumour content.

Prediction analysis was initially undertaken on the full set of 43 biopsies. The optimum misclassification error estimate (LOOCV) for the whole sample set was 28% using a three-gene predictor (Figure 1). To explore the effect of biopsy tumour content (and its associated influence on the expression profile) on error of response prediction, we selected three overlapping subsets of tumour samples which were more homogeneous in terms of percentage tumour content: (a) ≥ 50% (50%–95%) invasive tumour (25 samples), (b) ≤ 50% (20%–50%) invasive tumour (24 samples), and (c) 35%–60% invasive tumour (24 samples).

The subset consisting of 35%–60% represented a group centered around the median sample in terms of percentage tumour content and of a size similar to or the same as the other two groups.

The minimum error of classification for each of the subsets (8%–13%) was lower than that for the superset of all samples (28%) (Figure 1), suggesting that homogeneity of tumour content rather than tumour content *per se *might be an important factor for response prediction. However, the comparison of the subset error estimates with that for all samples is not controlled for the different sample sizes involved. To address this, we determined a 'subset permutation' *p *value for each of the subsets (see Materials and methods). We also estimated the probability that the subset errors arose by chance (a 'label permutation' as discussed in Materials and methods). Error estimates and corresponding *p *values for the 'subset permutation' and the 'label permutation' support the hypothesis that homogeneity of biopsy tumour content improves response prediction with the nearest neighbour algorithm (Table 3). The identity of the genes used in the prediction for each of these subgroups is presented in Table 4. The mean differential expression between good and poor responders for these genes is given in the supplementary information (Additional file 5).

We found, in common with other studies [20,22,30-32], that ER-positive and ER-negative tumours were very distinct in molecular terms (Additional file 4), which may confound response prediction. Therefore, response prediction was performed on ER-positive samples alone (16 good, 13 poor responders). Error rates, however, were high (31% with 8 genes, 31% with 25 genes). Within this subset, the effect of heterogeneity of tumour content on prediction appeared to be more pronounced (error rates: 31% (all samples, *n *= 29) versus 4.5% (≤ 60% tumour, *n *= 22)).

### Incorporation of histological information into the predictor

To explore further the effect of biopsy composition on prediction error, we attempted to create a single predictor for all samples by adding information about tumour content to the predictor as an extra dimension or 'histology gene'. The impact of variation in biopsy tumour content on prediction error was thought to be due partly to the fact that genes that discriminate between good and poor responders in high-tumour-content biopsies are often poor discriminators in low-tumour-content biopsies (and vice versa) as is evident from Table 4. To overcome this complication, 10 genes were selected that showed 100% support in the subset permutations (Table 4). By adding the proportion of tumour content to these 10 genes as an 11th dimension, we aimed to move the samples apart in 'prediction space' such that differences in tumour content also contributed to the distances between samples. Thus, samples with similar expression profiles become nearest neighbours of similar proportion of tumour content as well. In the nearest neighbour predictor, like is matched with like in terms of proportion of tumour content as well as expression profile.

**Figure 2:**
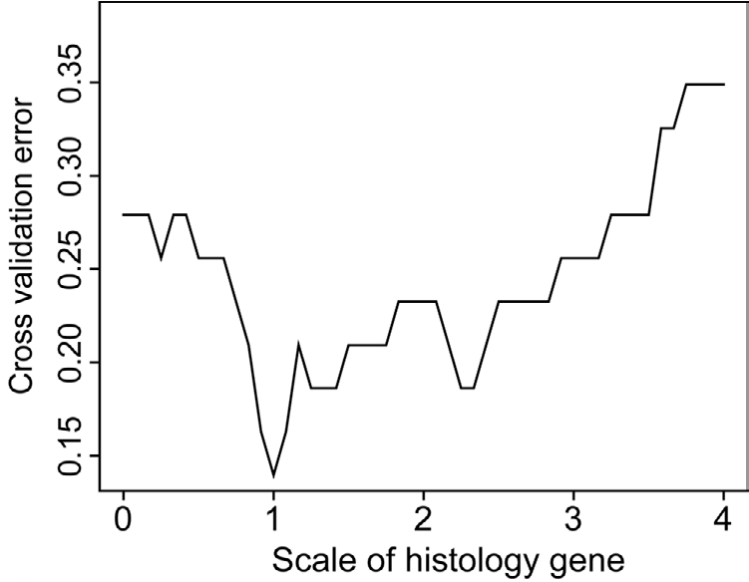
Variation in error rate according to scale of 'histology gene'. An 11th ('histology') gene was added to a 10-gene predictor as an extra dimension. This gene was rescaled from 0 to 4 standard deviations, and the effect on cross-validation error calculated.

It was necessary to rescale the histology gene's contribution to the distance between samples because the standard deviation of most genes lay between 0.5 and 1.5 (log_2 _ratio scale). Therefore, the percentage tumour figures were standardised by subtracting the average and dividing by the standard deviation. The 'histology gene' or 11th dimension was then rescaled from 0 to 4 standard deviations, and the effect on cross-validation error determined (Figure 2). After an initial drop in error, as the scale approached 1.0, the error rose beyond the initial error as the influence of the histology gene became too dominant. To test whether the effect of the histology gene was specific to the particular set of 10 genes used above, we tested the effect of adding the histology gene to 1,000 predictors, each containing a random permutation of between 8 and 16 genes drawn from the top 20 genes ranked by a combined score (Additional file 6 a-c). In 91% of these, the addition of the histology gene resulted in a lower error; in 49%, the error drop was greater than 10%.

## Discussion

In this study, we set out to explore the possibility that the transcriptional profile(s) of breast tumours relates to sensitivity to neoadjuvant chemotherapy. Such a profile might be useful in understanding the molecular mechanisms determining response or resistance and would provide the basis for a predictor of chemotherapy response.

However, the biopsy material used in this study had a complex cellular composition. In planning this study, consideration had been given to two approaches for ensuring that the percentage invasive tumour within biopsies was 'sufficient' and homogeneous. Cell selection, either by gross dissection or laser capture microdissection [33], allows enrichment of the invasive tumour component. We chose, in common with others [6,7,11,13,14,31], to set a minimum threshold for 'percentage malignant cells' within a given biopsy. The median percentage invasive tumour for the core biopsy samples was 50%, and the range of figures that were included in the study was 20% to 95%. In many biopsies, the dominant non-tumour component, connective tissue, was admixed with epithelial components, making enrichment of the malignant compartment difficult using gross dissection alone.

We hypothesised that this 'contamination' of biopsies by significant and variable amounts of non-tumour components might confound tumour classification. Indeed, at least 10% of the genes (144 genes) remaining after preprocessing of the data were found to correlate with cellular composition using a SAM regression analysis. Genes relatively overexpressed in low-percentage tumours included established stromal-related genes (Table 2) (for example, Collagen (type XV, alpha-1) and Cadherin 5 (type 2, VE-cadherin)). The exact source (histological compartment) of production of a given RNA could be further confirmed by FISH (fluorescent *in situ *hybridisation) or by comparing gene expression in microdissected stromal and tumour compartments.

However, paired biopsies, despite differences in proportional non-tumour content (Additional file 3), co-aggregated on cluster analysis (Additional file 1), suggesting a dominant 'tumour profile' despite variation in the proportion of stroma. Furthermore, prediction of ER status was not confounded by marked variation in percentage tumour content. This is in keeping with a number of studies that have shown that strong differential expression of a relatively high proportion of genes correlates with ER status [6,7,20,30-32] which would be expected to result in domination of the expression profile despite variation in the contribution by the non-tumour component. Furthermore, it has not been demonstrated that the ER expression signature is derived entirely from tumour cells. However, variation in the proportion of stroma may be sufficient to mask more subtle aspects of the tumour expression signature.

We found that the error rate for response prediction for the whole sample set was poor (28%) but was improved by increasing the homogeneity of cellular composition by subsetting on the basis of histological composition (error rate, 8%–13%) (Table 3). The misclassification rates for the subsets were determined with LOOCV and therefore represent high variance estimates, and the sample numbers are modest. We have used permutation analysis as support for the error estimates; ultimately, however, validation with an independent data set would address these issues.

We did not find any evidence to suggest that highly stromal biopsies result in higher prediction error. However, stromal-tumour content appears to affect the selection of genes that are used in the predictor for each histological subset, resulting in different but overlapping lists of predictive genes (Table 4). Some genes discriminated response in the 'high percentage' and not the 'low percentage' samples (for example, *PBEF1*). This may simply be a dose effect whereby discriminatory tumour-associated genes are no longer differential in 'low percentage samples' due to low signal. Alternatively, discriminatory genes that are expressed in both tumour and non-tumour compartments may lose discriminatory potential in tumours with a significant stromal contribution to the molecular signature. Genes that are discriminatory in low but not high 'percentage samples' (for example, *SOD1*) could be expressed only at the tumour-stroma interface in stromal and/or tumour cells. Certainly, breast tumour-induced changes in stromal expression have been previously documented [34]. Furthermore, it has been shown that tumour gene upregulation can occur specifically at the tumour-stroma interface [35,36]. Finally, it is also likely that the volume and configuration of stromal tissue within a tumour are a reflection of the tumour molecular subtype.

Thus, this analysis resulted in three distinct response-prediction genes lists that partially overlapped. *PBEF*, reported in one study to act as an inhibitor of apoptosis in neutrophils [37], appeared in the predictor for response for 'high percentage' tumours and has also been reported as a component of a three-gene predictor of AC sensitivity by another group [13]. In both studies, expression of this gene was higher in the resistant tumours than in sensitive tumours. *SOD1 *was found to be relatively overexpressed in resistant tumours ('low' and 'mid percentage') in keeping with a proposed role in the neutralisation of free radicals, one means by which anthracyclines are thought to inflict cellular damage [38]. *NDRG1*, upregulated in poor responders ('high percentage'), is induced by hypoxia and may reduce *p53 *expression [39]. Hypoxia may be a marker of poor vascularisation of tumours and therefore possible limitation of drug access.

Although here we have defined histological subsets, we have shown that it might be feasible to build a predictor that extracts information about the cellular composition of the biopsy from expression data. We found that adding standardised percentage tumour values as a 'histology gene' (Figure 2) to multigene predictors (Table 4) reduced the error rate significantly, supporting the idea that it may be possible to devise a predictor that operates regardless of biopsy composition. This would avoid the need for microdissection to enrich for malignant cells. Furthermore, if stromal or stromal-interface gene expression does carry discriminatory information, then microdissection would result in loss of predictive information.

Several groups have attempted to define a multigene predictor of chemoresponsiveness [10-15], using either clinical or pathological definitions of response. The reported error rates in these studies, as assessed on an independent data set or by LOOCV, range from 5% to 30%. Comparison of gene lists and error rates across these studies and with ours is hampered by the fact that the treatment regimens, response definitions, and microarray platforms differ and that the histological composition of the samples in most studies was not presented.

A number of samples used in our study have also been profiled using an Affymetrix platform (Santa Clara, CA, USA) [40]. In this independent analysis, 12 samples from tumours displaying either cCR or pCR and six samples from tumours with residual tumour greater than 70% were used to define a classifier (samples with less than 40% tumour were excluded from the study). The error rate of prediction on LOOCV was 33% (*p *= 0.4 on permutation).

Therefore, to date, the reported error rates associated with expression array prediction of response, certainly for anthracycline combination chemotherapy, remain too high for clinical utility. This may be due in part to the fact that breast cancer is an extremely complex and heterogeneous disease that operates multiple mechanisms of chemotherapeutic response and resistance which are not consistent across different subtypes, particularly in the case of combination regimens incorporating agents that act by multiple mechanisms. Such complexities mandate that study designs be optimised in terms of biopsy quality and sample size.

## Conclusion

The response prediction using all pre-treatment biopsies was modestly effective. However, the percentage of invasive cancer cells within a sample influenced the expression profile. Response prediction on subsets of samples more homogeneous in terms of cellular composition was associated with lower error rates. We believe that it is essential that consideration be given to biopsy composition in planning future studies of this type using methods such as those discussed above. Larger studies are required to establish whether optimal accuracy of response prediction may be achieved by the development of profiles specific to immunohistochemical breast cancer subtypes.

## Abbreviations

AC = adriamycin and cyclophosphamide; cCR = complete clinical response; ER = oestrogen receptor; LOOCV = leave-one-out cross-validation; MRD = minimal residual disease; NC = no change; OCT = optimum cutting temperature embedding compound; pCR = complete pathological response; PR = partial response; RMH = Royal Marsden Hospital; SAM = significance analysis of microarrays.

## Competing interests

The authors declare that they have no competing interests.

## Authors' contributions

SC was involved in design of study, acquisition of data, analysis and interpretation of results, and drafting the manuscript and preparing the final version (Breakthrough Breast Cancer Research Centre). TP participated in data acquisition and the conception and design of the study (RMH). TD was involved in analysis and interpretation of results, and drafting the manuscript and preparing the final version (Breakthrough Breast Cancer Research Centre). LF contributed to data acquisition (Breakthrough Breast Cancer Research Centre). AM performed data acquisition and analysis (Breakthrough Breast Cancer Research Centre). IS participated in data acquisition (RMH). HV performed data analysis (Breakthrough Breast Cancer Research Centre). AA participated in the design of study, interpretation of results, and preparing the final version of the manuscript (Breakthrough Breast Cancer Research Centre). MD was involved in the conception and design of the study, interpretation of results, and preparing the final version of the manuscript (RMH). All authors read and approved the final manuscript.

## Supplementary Material

Additional file 1 A PDF file containing a dendrogram of flexible beta clustering with Spearman rank correlation on all core biopsy samples taken from 43 patients. Click here for file

Additional file 3 A word document containing a table that shows the characteristics of paired (repeat) core biopsy samples. Click here for file

Additional file 4 A word document containing a table that shows the oestrogen receptor (ER) predictive genes. Click here for file

Additional file 5 A word document containing a table that shows response prediction gene lists with expression ratios. Click here for file

Additional file 6 A PDF file showing Permutation to assess the generalisability of the reduction in error rate observed by addition of the 'histology gene'. Click here for file

Additional file 2 A word document containing the legend for additional data file 1. Click here for file

Additional file 7 A word document containing the legend for Additional data file 6. Click here for file

## Figures and Tables

**Table 1:** Patient, tumour, and pre-treatment biopsy characteristics (43 samples used in prediction analysis)

Age: median (range)	49 (28–68)				
Menopausal status	pre: 25 (58%)	post: 9 (21%)	na: 1 (2%)	HRT: 8 (19%)	
Histological subtype	lob: 6 (14%)	duct: 34 (79%)	duct/lob: 1 (2%)	medull: 1 (2%)	na: 1
ER status	+: 29 (67%)	-: 13 (30%)	na: 1 (2%)		
HER-2 status	+: 5 (12%)	-: 36 (84%)	na: 2 (5%)		
Inflamm infiltrate	nil: 19 (44%)	mild: 16 (37%)	mod: 6 (14%)	severe: 2 (5%)	
Grade	2: 18 (42%)	3: 24 (56%)	na: 1 (2%)		
Percentage invasive tumour: median (range)	50 (20–95)				
Response	pCR: 4 (9%)*	cCR: 16 (37%)	MRD: 3 (7%)	PR: 8 (19%)	NC: 12 (28%)
(*7 [16%] had no surgery)	good: 23 (53%)			poor: 20 (47%)	
Clinical stage:					
Primary dimension (*n*)					
2.0–3.0 cm	15				
3.1–4.0 cm	7				
4.1–5.0 cm	9				
>5.0 cm	11				
Multifocal	1				
Median dimension (range) (cm)	4 (2–10)				
			ER +	ER -	
Skin involvement (*n*)	2	HER2 +	4 (10%)	1 (2%)	
Inflammatory (*n*)	2				
		HER2 -	24 (56%)	12 (28%)	
Nodal involvement (*n*)				(2 na)	
N0	29				
N1	14				
N2	0				
NA	0				

'Postmenopausal' refers to patients not on HRT at diagnosis. Postmenopausal patients on HRT at diagnosis are listed under 'HRT'. 'Primary dimension' refers to the maximum tumour diameter (cm). 'Inflammatory infiltrate' score and 'median percentage invasive tumour' relate to values for the core used in prediction analysis. +, positive; -, negative; cCR, complete clinical response; duct, ductal; duct/lob, mixed ductal/lobular carcinoma; ER, oestrogen receptor; HRT, hormone replacement therapy; lob, lobular; medull, medullary; inflamm, inflammatory; mod, moderate; MRD, minimal residual disease; na, not available; NC, no change; pCR, complete pathological response; PR, partial response.

**Table 2:** SAM (significance analysis of microarrays) analysis of genes correlated to percentage tumour content.

**Gene name**	**Genbank accession**	**Unigene cluster ID**	**Symbol**	**Score (d)**	***q*-value (%)**
ADP-ribosylation factor 1	AK023803	Hs.286221	*ARF1*	**3.11**	**4.13**
CDC28 protein kinase regulatory subunit 2	BQ898943	Hs.83758	*CKS2*	**2.73**	**4.13**
TRK-fused gene	NM_006070	Hs.446568	*TFG*	**2.65**	**4.13**
Discs, large homolog 7 (*Drosophila*)	NM_014750	Hs.77695	*DLG7*	**2.60**	**4.13**
RAB6B, member RAS oncogene family	NM_016577	Hs.352530	*RAB6B*	**2.59**	**4.13**
					
Epithelial cell transforming sequence 2 oncogene	AY376439	Hs.293257	*ECT2*	**2.56**	**4.13**
					
FXYD domain containing ion transport regulator 2	CR627143	Hs.413137	*FXYD2*	**2.54**	**4.13**
					
Ubiquitin specific protease 9, X-linked (fat facets-like, *Drosophila*)	NM_004652	Hs.77578	*USP9X*	**2.48**	**4.13**
Pitrilysin metalloproteinase 1	CR749279	Hs.528300	*PITRM1*	**2.46**	**4.13**
					
Myeloid differentiation primary response gene (88)	AK124685	Hs.82116	*MYD88*	**2.44**	**4.13**
Baculoviral IAP repeat-containing 5 (survivin)	NM_001012271	Hs.1578	*BIRC5*	**2.42**	**4.13**
Polo-like kinase 4 (*Drosophila*)	NM_014264	Hs.172052	*PLK4*	**2.40**	**4.13**
2',3'-cyclic nucleotide 3' phosphodiesterase	NM_033133	Hs.443836	*CNP*	**2.40**	**4.13**
Triosephosphate isomerase 1	BM913099	Hs.83848	*TPI1*	**2.39**	**4.13**
ALL1-fused gene from chromosome 1q	BX649117	Hs.75823	*AF1Q*	**2.37**	**4.13**
					
Latent transforming growth factor beta binding protein 2	NM_000428	Hs.105689	*LTBP2*	-3.49	1.00
LIM domain binding 2	AL832772	Hs.4980	*LDB2*	-3.43	1.00
Collagen, type XV, alpha 1	NM_001855	Hs.409034	*COL15A1*	-3.39	1.00
					
Cadherin 5, type 2, VE-cadherin (vascular epithelium)	NM_001795	Hs.76206	*CDH5*	-3.39	1.00
Forkhead box O1A (rhabdomyosarcoma)	NM_002015	Hs.170133	*FOXO1A*	-3.17	1.00
					
TEK tyrosine kinase, endothelial (venous malformations, multiple cutaneous)	BC035514	Hs.89640	*TEK*	-3.02	1.00
Regulator of G-protein signaling 5	NM_003617	Hs.24950	*RGS5*	-2.97	1.00
					
Fasciculation and elongation protein zeta 1 (zygin I)	NM_022549	Hs.79226	*FEZ1*	-2.90	1.00
Vascular endothelial growth factor C	NM_005429	Hs.79141	*VEGFC*	-2.90	1.00
					
Tumor necrosis factor receptor superfamily, member 10c, decoy without an intracellular domain	NM_003841	Hs.253829	*TNFRSF10C*	-2.82	1.00
CDNA FLJ25106 fis, clone CBR01467	AK128814	Hs.432914		-2.79	1.00
Alpha-2-macroglobulin	CR749334	Hs.74561	*A2M*	-2.77	1.00
					
Meis1, myeloid ecotropic viral integration site 1 homolog 2 (mouse)	NM_170677	Hs.362805	*MEIS2*	-2.71	1.00
Tropomodulin 1	AK096156	Hs.404289	*TMOD1*	-2.70	1.00
Isoleucine-tRNA synthetase	NM_013417	Hs.172801	*IARS*	-2.63	1.00

Positive values for score (bold) indicate positive correlation with percentage tumour, and negative values indicate inverse correlation with percentage tumour (underlined). The table has been truncated to show only genes with the lowest false discovery rates (q).

**Table 3:** Misclassification error estimates (leave-one-out cross-validation) for response prediction.

**Set of samples**	**Sample size**	**Minimum error rate**	***n *of genes used in predictor**	***p *value on 'subset' permutation**	***p *value on 'label' permutation**
All (20%–90%)	43	28%	3	Not determined	0.079
High range (50%–95%)	25	12%	4	0.026	0.021
Low range (20%–50%)	24	13%	7	0.039	0.028
Mid range (35%–60%)	24	8%	7	0.001	0.002

Column 1 shows the division of samples into three groups according to percentage invasive tumour content. Details of minimum error predictors for all samples and subsets. Permutation *p *values were calculated as described in Materials and methods.

**Table 4:** Response prediction gene lists.

**Symbol**	**Genbank accession**	**Unigene cluster ID**	**All**	**Low**	**Mid**	**High**	**Name**
*ZNF146*	NM_007145	Hs.444223	100	100	100	0	Zinc finger protein 146
*RFC3*	CR749565	Hs.115474	100	12	100	0	Replication factor C (activator 1) 3, 38kDa
*DLD*	NM_000108	Hs.74635	100	4	100	0	Dihydrolipoamide dehydrogenase
*EIF3S8*	NM_003752	Hs.534402	0	100	58	0	Eukaryotic translation initiation factor 3, subunit 8
*SOD1*	BF131654	Hs.443914	0	91	25	0	Superoxide dismutase 1, soluble (amyotrophic lateral sclerosis 1 (adult))
*PBEF1*	NM_005746	Hs.293464	25	0	0	88	Pre-B-cell colony enhancing factor 1
*CD9*	AK025016	Hs.387579	0	100	0	0	CD9 antigen (p24)
*TCEB1*	NM_005648	Hs.435169	0	100	0	0	Transcription elongation factor B (SIII), polypeptide 1 (15kDa, elongin C)
*IGFBP4*	CR603382	Hs.1516	0	0	100	0	Insulin-like growth factor binding protein 4
*STMN1*	BX647885	Hs.209983	0	0	100	0	Stathmin 1/oncoprotein 18
*NDRG1*	AK124709	Hs.318567	0	0	0	100	N-myc downstream regulated gene 1
*PAM*	NM_000919	Hs.352733	0	0	0	100	Peptidylglycine alpha-amidating monooxygenase
*ARHE*	X97758	Hs.6838	20	0	0	76	Ras homolog gene family, member E
*MAPK6*	NM_002748	Hs.271980	95	0	0	0	Mitogen-activated protein kinase 6
*SFRS11*	BX640645	Hs.443458	0	91	0	0	Splicing factor, arginine/serine-rich 11
*TESK1*	BC038448	Hs.79358	79	0	0	0	Testis-specific kinase 1
*THRAP3*	NM_005119	Hs.250113	60	0	0	0	Thyroid hormone receptor associated protein 3
*PTPN7*	AK127214	Hs.402773	55	0	0	0	Protein tyrosine phosphatase, non-receptor type 7
*PSMC6*	AK127456	Hs.156171	0	41	0	0	Proteasome (prosome, macropain) 26S subunit, ATPase, 6
*CD47*	NM_001777	Hs.446414	0	0	37	0	CD47 antigen (Rh-related antigen, integrin-associated signal transducer)
*REST*	NM_005612	Hs.401145	23	0	12	0	RE1-silencing transcription factor
*PCNA*	NM_002592	Hs.78996	0	0	33	0	Proliferating cell nuclear antigen
*CTPS*	BC009408	Hs.251871	0	0	16	0	CTP synthase
*ARL6IP*	BC010281	Hs.75249	0	12	0	0	ADP-ribosylation factor-like 6 interacting protein
*CDH1*	NM_004360	Hs.194657	0	12	0	0	Cadherin 1, type 1, E-cadherin (epithelial)
*ITSN1*	AF064244	Hs.66392	0	0	0	12	Intersectin 1 (SH3 domain protein)
*GATA4*	NM_002052	Hs.243987	9	0	0	0	GATA binding protein 4
*SNRPB*	BM564070	Hs.83753	0	8	0	0	Small nuclear ribonucleoprotein polypeptides B and B1
*DLG7*	NM_014750	Hs.77695	0	0	8	0	Discs, large homolog 7 (Drosophila)
*KCNN3*	BX649146	Hs.89230	0	0	0	8	Potassium intermediate/small conductance calcium-activated channel

The genes used in the prediction for the full set of samples ('all') and for each of the subsets (defined in Table 3). For each subset, the genes listed are those used in the minimum error rate predictor (except for the 'all' category, for which a seven-gene predictor was selected to observe a longer gene list to allow comparison with the other gene lists). The 'support' given for each gene refers to the percentage of the leave-one-out cross-validations in which the gene was used. Only genes that attain cumulative support across 'all' and the subsets of at least 8% are shown.
