# Supplementary figures and images for: The effect of the stromal component of breast tumours on prediction of clinical outcome using gene expression microarray analysis
Source: Breast Cancer Res. 2006 Jun 21;8(3):R32. doi: 10.1186/bcr1506 (PMC1557729; doi:10.1186/bcr1506)

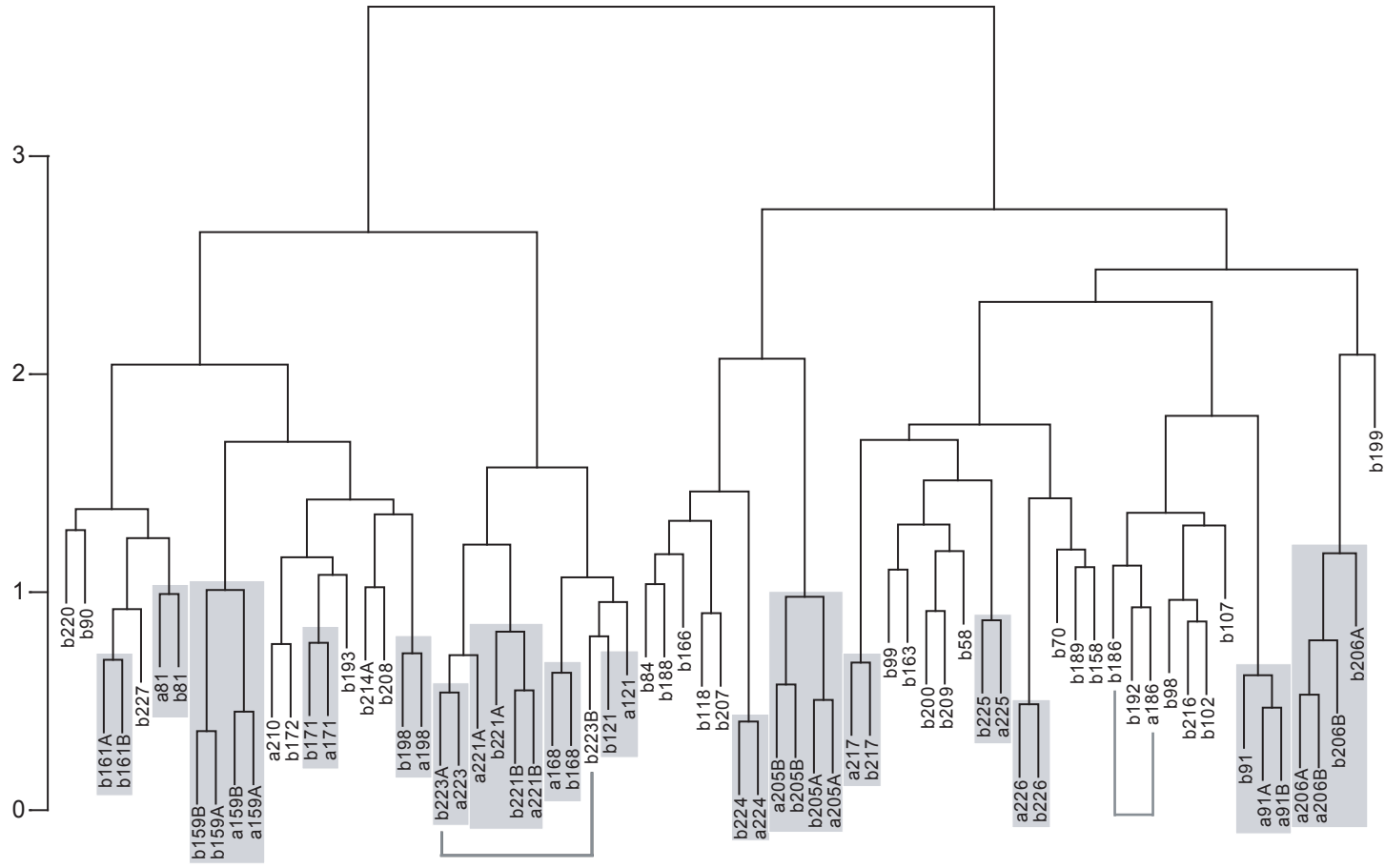

Supplement: Additional file 1 — A PDF file containing a dendrogram of flexible beta clustering with Spearman rank correlation on all core biopsy samples taken from 43 patients. [file bcr1506-S1.pdf]

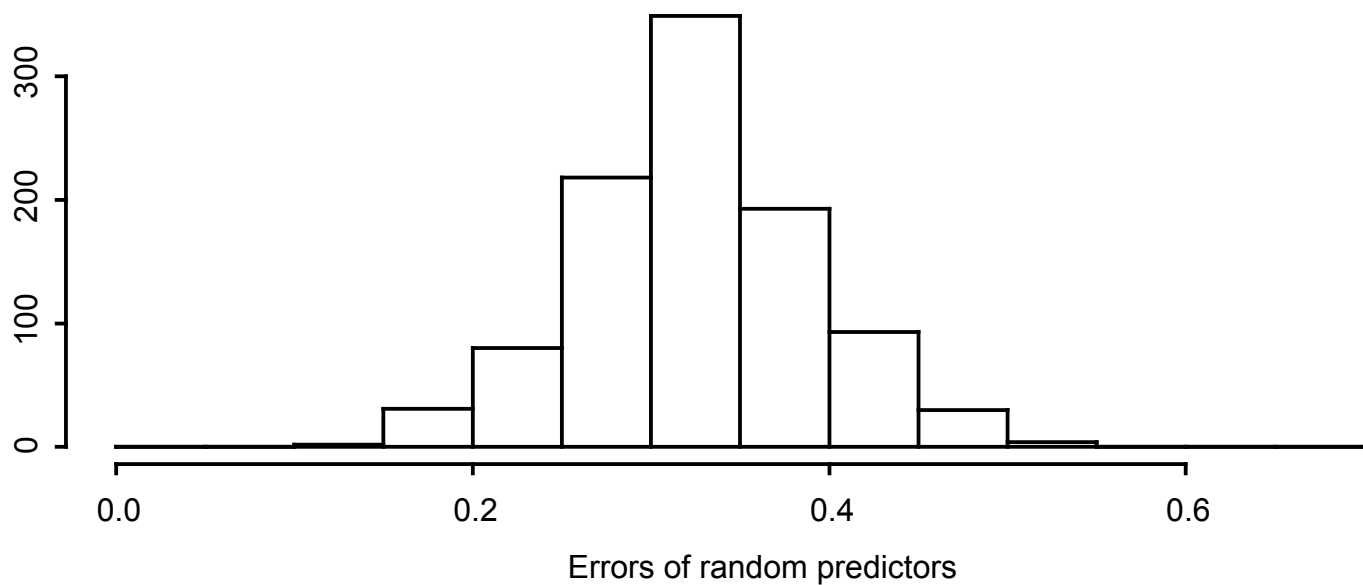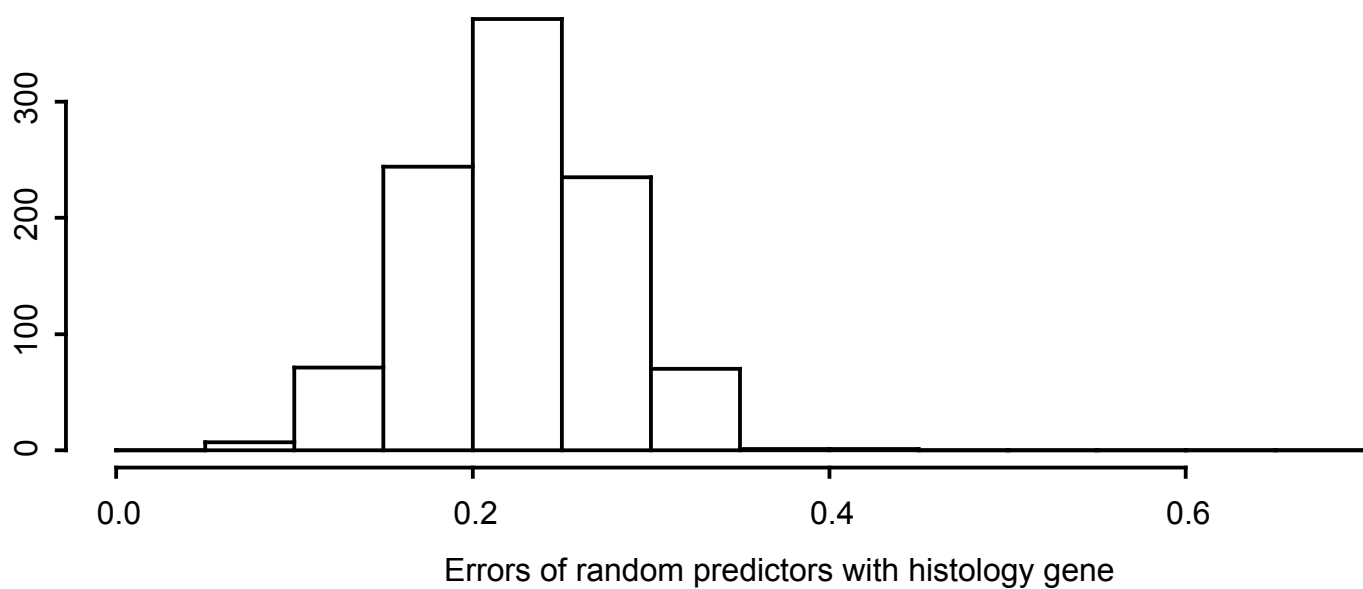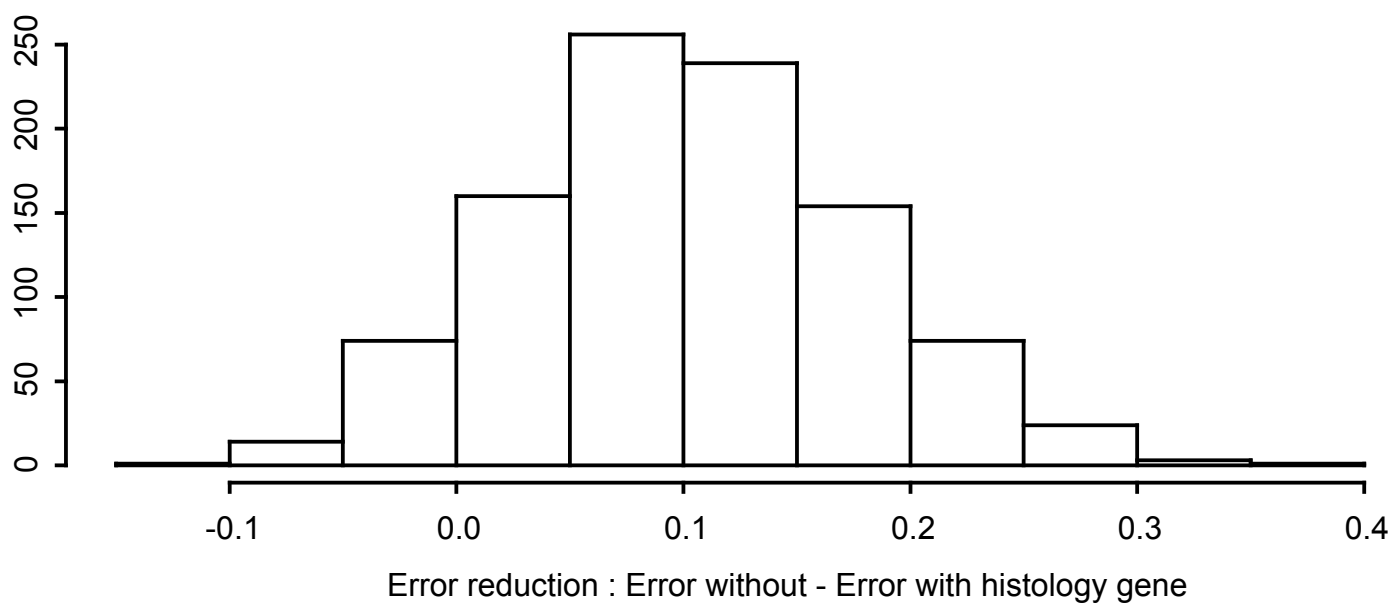

Supplement: Additional file 6 — A PDF file showing Permutation to assess the generalisability of the reduction in error rate observed by addition of the 'histology gene'. [file bcr1506-S6.pdf]
