# Supplementary material for: The effect of the stromal component of breast tumours on prediction of clinical outcome using gene expression microarray analysis
Source: Breast Cancer Res. 2006 Jun 21;8(3):R32. doi: 10.1186/bcr1506 (PMC1557729; doi:10.1186/bcr1506)
Supplement: Additional file 3 — A word document containing a table that shows the characteristics of paired (repeat) core biopsy samples. [file bcr1506-S3.doc]

| Characteristics of duplicate (repeat) core-biopsy samples | | | | | |
| --- | --- | --- | --- | --- | --- |
| **pt study no.** | **sample no.** | **pre/ post** | **inflamm infil** | **% invasive** | **Normal (y/n)** |
| **206** | b206A | pre | mild | 45 | n |
| **206** | b206B | pre | nil | 50 | y |
| **206** | a206A | post | mild | 20 | y |
| **206** | a206B | post | mild | 30 | n |
| **91** | a91A | post | mild | 60 | n |
| **91** | a91B | post | mild | 50 | y |
| **205** | b205A | pre | mod | 50 | n |
| **205** | b205B | pre | mild | 60 | n |
| **205** | b205A | post | mod | 60 | n |
| **205** | b205B | post | mod | 50 | n |
| **159** | a159A | post | mild | 45 | y |
| **159** | a159B | post | nil | 30 | y, 30% |
| **161** | b161A | pre | mild | 35 | y |
| **161** | b161B | pre | mod | 20 | n |
| **221** | b221A | pre | nil | 50 | n |
| **221** | b221B | pre | mild | 40 | y |
| **221** | a221A | post | mod | 30 | y, 30% |
| **221** | a221B | post | nil | 50 | 0 |
| **223** | b223A | pre | nil | 30 | 0 |
| **223** | b223B | pre | nil | 20 | 0 |

Assessment of inflammatory infiltrate, presence of normal epithelial cells and percentage invasive tumour was made on the lowest level. ‘Pre/post’ refers to the timing relative to treatment. ‘Normal’ refers to normal epithelial cells and unless otherwise specified the amount present was minimal.

Abbreviations: pt, patient; inv, invasive; y, present; n, absent; inflamm, inflammatory; infil, infiltrate; mod, moderate.
