# Supplementary material for: The effect of the stromal component of breast tumours on prediction of clinical outcome using gene expression microarray analysis
Source: Breast Cancer Res. 2006 Jun 21;8(3):R32. doi: 10.1186/bcr1506 (PMC1557729; doi:10.1186/bcr1506)
Supplement: Additional file 4 — A word document containing a table that shows the oestrogen receptor (ER) predictive genes. [file bcr1506-S4.doc]

| **ER predictive genes** | | | | | |
| --- | --- | --- | --- | --- | --- |
| **Genbank Accession** | **Unigene Cluster ID** | **Support** | **gene name** | **gene symbol** | **expression ratio neg.by.pos** |
| AK126153 | Hs.26010 | 42 | Phosphofructokinase, platelet | PFKP | 1.71 |
| BC005856 | Hs.36761 | 42 | HRAS-like suppressor | HRASLS | 1.44 |
| CR616919 | Hs.406013 | 42 | Keratin 18 | KRT18 | -1.34 |
| NM_004311 | Hs.182215 | 42 | ADP-ribosylation factor-like 3 | ARL3 | -0.81 |
| AK128739 | Hs.128686 | 42 | Nucleobindin 2 | NUCB2 | -1.17 |
| AK172792 | Hs.130989 | 42 | Sodium channel, nonvoltage-gated 1 alpha | SCNN1A | -1.85 |
| D87076 | Hs.397990 | 42 | PHD finger protein 15 | PHF15 | -0.97 |
| BX537451 | Hs.83532 | 42 | Membrane cofactor protein (CD46, trophoblast-lymphocyte cross-reactive antigen) | MCP | -0.96 |
| NM_006184 | Hs.434924 | 42 | Regulating synaptic membrane exocytosis 3 | RIMS3 | 1.09 |
| AK126963 | Hs.406050 | 42 | Dynein, axonemal, light intermediate polypeptide 1 | DNALI1 | -1 |
| BC041846 | Hs.191842 | 42 | Cadherin 3, type 1, P-cadherin (placental) | CDH3 | 1.13 |
| BC040989 | Hs.170160 | 42 | Ral guanine nucleotide dissociation stimulator-like 2 | RGL2 | -0.76 |
| AL157440 | Hs.74120 | 42 | Chromosome 10 open reading frame 116 | C10orf116 | -2.09 |
| NM_012319 | Hs.79136 | 42 | Solute carrier family 39 (zinc transporter), member 6 | SLC39A6 | -2.26 |
| NM_004496 | Hs.163484 | 42 | Forkhead box A1 | FOXA1 | -2.28 |
| BM923753 | Hs.350470 | 42 | Trefoil factor 1 (breast cancer, estrogen-inducible sequence expressed in) | TFF1 | -3.79 |
| CR591371 | Hs.695 | 42 | Cystatin B (stefin B) | CSTB | 0.61 |
| BF667541 | Hs.397062 | 42 | Peroxiredoxin 3 | PRDX3 | -0.96 |
| NM_000216 | Hs.380850 | 42 | Kallmann syndrome 1 sequence | KAL1 | -1.01 |
| NM_001552 | Hs.1516 | 42 | Insulin-like growth factor binding protein 4 | IGFBP4 | -1 |
| BC009408 | Hs.251871 | 42 | CTP synthase | CTPS | 0.85 |
| NM_016267 | Hs.9030 | 42 | Vestigial like 1 (Drosophila) | VGLL1 | 2.67 |
| CR620277 | Hs.406504 | 42 | Transgelin 2 | TAGLN2 | 0.88 |
| AK090727 | Hs.59106 | 42 | Cell growth regulator with ring finger domain 1 | CGRRF1 | -0.63 |
| NM_001005414 | Hs.42650 | 42 | ZW10 interactor | ZWINT | -1.21 |
| NM_006670 | Hs.250822 | 42 | Serine/threonine kinase 6 | STK6 | -1.34 |
| AF467287 | Hs.209846 | 42 | LPS-responsive vesicle trafficking, beach and anchor containing | LRBA | -1.23 |
| AB208815 | Hs.26770 | 42 | Fatty acid binding protein 7, brain | FABP7 | 2.48 |
| NM_000633 | Hs.79241 | 42 | B-cell CLL/lymphoma 2 | BCL2 | -1.3 |
| BM924855 | Hs.367992 | 42 | Inositol(myo)-1(or 4)-monophosphatase 2 | IMPA2 | 0.95 |
| AK091962 | Hs.84753 | 42 | Hypothetical protein FLJ12442 | FLJ12442 | 1.01 |
| NM_016835 | Hs.101174 | 42 | Microtubule-associated protein tau | MAPT | -1.47 |
| BX537826 | Hs.446567 | 42 | Basic transcription factor 3 | BTF3 | -0.9 |
| AF040709 | Hs.270804 | 42 | CREB binding protein (Rubinstein-Taybi syndrome) | CREBBP | -0.71 |
| AK095781 | Hs.515010 | 42 | Cold inducible RNA binding protein | CIRBP | -0.99 |
| NM_001002295 | Hs.169946 | 42 | GATA binding protein 3 | GATA3 | -1.01 |
| NM_004538 | Hs.21365 | 42 | 1-like 3 | NAPL3 | -0.83 |
| BX537826 | Hs.446567 | 42 | Basic transcription factor 3 | BTF3 | -0.91 |
| BX649090 | Hs.153687 | 42 | Inositol polyphosphate-4-phosphatase, type II, 105kDa | INPP4B | -1.19 |
| BC039247 | Hs.218182 | 42 | RUN domain containing 1 | RUNDC1 | -0.77 |
| NM_001219 | Hs.7753 | 42 | Calumenin | CALU | 0.87 |
| NM_003046 | Hs.448520 | 42 | Solute carrier family 7 (cationic amino acid transporter, y+ system), member 2 | SLC7A2 | -1.71 |
| NM_002444 | Hs.170328 | 42 | Moesin | MSN | 0.66 |
| D86962 | Hs.130119 | 42 | Growth factor receptor-bound protein 10 | GRB10 | 0.67 |
