# Supplementary material for: The effect of the stromal component of breast tumours on prediction of clinical outcome using gene expression microarray analysis
Source: Breast Cancer Res. 2006 Jun 21;8(3):R32. doi: 10.1186/bcr1506 (PMC1557729; doi:10.1186/bcr1506)
Supplement: Additional file 5 — A word document containing a table that shows response prediction gene lists with expression ratios. [file bcr1506-S5.doc]

| **Response prediction genes with expression ratios** | | | | | | |
| --- | --- | --- | --- | --- | --- | --- |
| **subgroup** | **Genbank accession** | **Unigene Cluster ID** | **Support** | **gene name** | **gene symbol** | **good.by.poor** |
| **High %t** |  |  |  |  |  |  |
|  | AK124709 | Hs.318567 | 25 | N-myc downstream regulated gene 1 | NDRG1 | -0.75 |
|  | NM_000919 | Hs.369430 | 25 | Peptidylglycine alpha-amidating monooxygenase | PAM | -0.80 |
|  | NM_005746 | Hs.489615 | 22 | Pre-B-cell colony enhancing factor 1 | PBEF1 | -0.64 |
|  | X97758 | Hs.6838 | 19 | Ras homolog gene family, member E | ARHE | -0.63 |
|  | AF064244 | Hs.66392 | 3 | Intersectin 1 (SH3 domain protein) | ITSN1 | -0.38 |
|  | BX649146 | Hs.490765 | 2 | Potassium intermediate/small conductance calcium-activated channel, subfamily N | KCNN3 | 0.90 |
|  | NM_001801 | Hs.442378 | 1 | Cysteine dioxygenase, type I | CDO1 | -0.71 |
|  | AB209433 | Hs.520038 | 1 | HLA-B associated transcript 8 | BAT8 | 0.76 |
|  | BM704055 | Hs.472185 | 1 | NADH dehydrogenase (ubiquinone) Fe-S protein 5, 15kDa (NADH-coenzyme Q reductase) | NDUFS5 | -0.67 |
|  | NM_002126 | Hs.196952 | 1 | Hepatic leukemia factor | HLF | -0.99 |
| **Low %** |  |  |  |  |  |  |
|  | NM_003752 | Hs.534402 | 24 | Eucariotic translation initiation factor 3, subunit 8, 110kDa | EIF3S8 | -0.94 |
|  | AK025016 | Hs.114286 | 24 | CD9 antigen (p24) | CD9 | -1.06 |
|  | BX647444 | Hs.444223 | 24 | Zinc finger protein 146 | ZNF146 | -0.80 |
|  | NM_005648 | Hs.533437 | 24 | Transcription elongation factor B (SIII), polypeptide 1 (15kDa, elongin C) | TCEB1 | -0.83 |
|  | BF131654 | Hs.443914 | 22 | Superoxide dismutase 1, soluble (amyotrophic lateral sclerosis 1 (adult)) | SOD1 | -0.85 |
|  | BX640645 | Hs.479693 | 22 | Splicing factor, arginine/serine-rich 11 | SFRS11 | 0.50 |
|  | BX356714 | Hs.156171 | 10 | Proteasome (prosome, macropain) 26S subunit, ATPase, 6 | PSMC6 | -0.91 |
|  | BC010281 | Hs.75249 | 3 | ADP-ribosylation factor-like 6 interacting protein | ARL6IP | -0.62 |
|  | CR749565 | Hs.115474 | 3 | Replication factor C (activator 1) 3, 38kDa | RFC3 | -0.87 |
|  | NM_004360 | Hs.461086 | 3 | Cadherin 1, type 1, E-cadherin (epithelial) | CDH1 | -1.46 |
|  | BM564070 | Hs.83753 | 2 | Small nuclear ribonucleoprotein polypeptides B and B1 | SNRPB | -0.49 |
|  | CR933675 | Hs.359289 | 1 | Transcription factor 4 | TCF4 | 0.69 |
|  | NM_000108 | Hs.131711 | 1 | Dihydrolipoamide dehydrogenase | DLD | -0.48 |
|  | NM_032105 | Hs.269777 | 1 | Protein phosphatase 1, regulatory (inhibitor) subunit 12B | PPP1R12B | 0.43 |
|  | BX648303 | Hs.396783 | 1 | Solute carrier family 9 (sodium/hydrogen exchanger), isoform 3 regulator 1 | SLC9A3R1 | -0.64 |
|  | NM_004381 | Hs.42853 | 1 | CAMP responsive element binding protein-like 1 | CREBL1 | -0.34 |
|  | NM_005197 | Hs.211773 | 1 | Checkpoint suppressor 1 | CHES1 | 0.49 |
|  | AB209219 | Hs.513288 | 1 | Zinc finger protein 200 | ZNF200 | 0.38 |
| **mid %** |  |  |  |  |  |  |
|  | NM_000108 | Hs.131711 | 24 | Dihydrolipoamide dehydrogenase | DLD | -0.54 |
|  | BX647444 | Hs.444223 | 24 | Zinc finger protein 146 | ZNF146 | -0.56 |
|  | NM_001552 | Hs.1516 | 24 | Insulin-like growth factor binding protein 4 | IGFBP4 | 0.74 |
|  | CR749565 | Hs.115474 | 24 | Replication factor C (activator 1) 3, 38kDa | RFC3 | -1.07 |
|  | BX647885 | Hs.209983 | 24 | Stathmin 1/oncoprotein 18 | STMN1 | -0.75 |
|  | NM_003752 | Hs.534402 | 14 | Eucariotic translation initiation factor 3, subunit 8, 110kDa | EIF3S8 | -0.77 |
|  | NM_001777 | Hs.446414 | 9 | CD47 antigen (Rh-related antigen, integrin-associated signal transducer) | CD47 | -0.78 |
|  | BC012609 | Hs.78996 | 8 | proliferating cell nuclear antigen | PCNA | -0.61 |
|  | BF131654 | Hs.443914 | 6 | Superoxide dismutase 1, soluble (amyotrophic lateral sclerosis 1 (adult)) | SOD1 | -0.68 |
|  | BC009408 | Hs.251871 | 4 | CTP synthase | CTPS | -0.59 |
|  | NM_005612 | Hs.401145 | 3 | RE1-silencing transcription factor | REST | 0.66 |
|  | NM_014750 | Hs.77695 | 2 | Discs, large homolog 7 (Drosophila) | DLG7 | -0.63 |
|  | CR933728 | Hs.334562 | 1 | Cell division cycle 2, G1 to S and G2 to M | CDC2 | -0.89 |
|  | BC041005 | Hs.1145 | 1 | Wilms tumor 1 | WT1 | -0.62 |

For each subgroup, the genes listed are those used when the error rate of prediction was minimal (**see Table 3)**. The expression data given relates to mean Log_2_ expression ratio for good/poor responders.
