# Supplementary material for: The effect of the stromal component of breast tumours on prediction of clinical outcome using gene expression microarray analysis
Source: Breast Cancer Res. 2006 Jun 21;8(3):R32. doi: 10.1186/bcr1506 (PMC1557729; doi:10.1186/bcr1506)
Supplement: Additional file 2 — A word document containing the legend for additional data file 1. [file bcr1506-S2.doc]

**Dendrogram of sample clustering**

Flexible beta clustering with Spearman rank correlation on all core-biopsy samples taken from 43 patients. The last digit of each label denotes a sample number and the preceding digits denote the patient number (code). ‘a’ or ‘b’ preceding a sample number indicates whether the sample was taken ‘after’ or ‘before’ therapy respectively. When duplicate samples were taken at the same time point these are denoted by ‘A’ or ‘B’. Samples from the same tumour are boxed in grey. The scale represents a ‘distance measure’. The only ‘same cancer’ samples not to co-segregate are b223A/a223 separated from b223B, and b186 separated from a186 (denoted by the 'U bars').
