# Supplementary material for: The effect of the stromal component of breast tumours on prediction of clinical outcome using gene expression microarray analysis
Source: Breast Cancer Res. 2006 Jun 21;8(3):R32. doi: 10.1186/bcr1506 (PMC1557729; doi:10.1186/bcr1506)
Supplement: Additional file 7 — A word document containing the legend for Additional data file 6. [file bcr1506-S7.doc]

**Permutation to assess the generalizability of the reduction in error rate observed by addition of the ‘histology gene’**

To assess whether the reduction seen in error rate was specific to the selected set of 10 discriminatory genes all 1280 genes were ranked by adding the squared values of the Kolmogorov Smirnov statistic (unweighted this time ie 0.0 to 1.0) from the subsets as described above. In this scoring system high scores in any single subset will effect the gene ranking more than medium scores across all subsets. From the 20 highest scoring genes random gene subsets consisting of between 8 and 16 genes were selected so that both the number of genes and the particular genes used varied.

For each of these predictors we determined the predictor error with and without the addition of the histology gene. Histograms of the errors are shown without (**S2a**) and with (**S2b**) the histology gene. The distribution of errors for these random predictors is shifted toward lower errors by incorporation of information about tumour content from the histology gene. Five of these predictors (all unique) gave errors of 9% (4 errors) when combined with the histology gene. **Fig. S2c** shows the histogram of paired error differences such that positive values indicate reduced error by addition of the histology gene. Of the 1000 predictors, 91% showed either no change or a fall in prediction error, 49% showed an absolute reduction in error rate of greater than 10% and 10% showed an absolute reduction in error rate of greater than 20%.
